# Effects of high intensity interval training (HIIT) on cardiopulmonary fitness and physical function in middle-aged and elderly women: a systematic review and meta-analysis

**DOI:** 10.3389/fphys.2026.1778052

**Published:** 2026-03-04

**Authors:** Limin Cai, Jintao Guo, Ruohan Zhang, Jinfa Gu, Longtao Zhao, Jianzhong Wu, Yueyang Yu, Si Chen

**Affiliations:** 1 Department of Physical Education, North China Electric Power University, Beijing, China; 2 College of Competitive Sports, Beijing Sport University, Beijing, China; 3 Exercise and Sports Science Programme, School of Health Sciences, Universiti Sains Malaysia, Kota Bharu, Kelantan, Malaysia; 4 College of Physical Education and Health Sciences, Zhejiang Normal University, Jinhua, Zhejiang, China

**Keywords:** high-intensity interval training, meta-analysis, older women, physical function, VO_2max_

## Abstract

**Background:**

To systematically evaluate the effects of high-intensity interval training (HIIT) on cardiorespiratory fitness and physical function in middle-aged and older women.

**Methods:**

PubMed, Web of Science, and Scopus were searched from inception to November 2025. Randomized controlled trials comparing HIIT with control interventions in middle-aged and older women were included. Random-effects meta-analyses were performed. Primary outcomes were maximal or peak oxygen uptake (VO_2max_/VO_2peak_) and physical or functional performance measures.

**Results:**

Nineteen randomized controlled trials were included. Meta-analysis showed that HIIT significantly improved VO_2max_ compared with control interventions (SMD = 1.20, 95% CI 0.86–1.54, I^2^ = 31%), with high certainty of evidence. No significant effect was observed for VO_2peak_ (SMD = 0.23, 95% CI −0.23 to 0.69). HIIT did not significantly improve muscle strength (SMD = −0.17, 95% CI −1.04 to 0.70), though strength assessments were not always specific to the muscle groups trained, flexibility, or sit-to-stand performance. Walking ability showed a borderline significant improvement (SMD = 0.49, 95% CI 0.00–0.97), with very low certainty of evidence. Subgroup analyses indicated consistent VO_2max_ improvements across age groups, body mass status, and intervention durations.

**Conclusion:**

HIIT significantly improves cardiorespiratory fitness in middle-aged and older women but shows limited effects on physical function. HIIT alone is insufficient to comprehensively improve functional performance.

**Systematic Review registration:**

https://www.crd.york.ac.uk/PROSPERO/view/CRD420251272861.

## 1 Introduction

Population aging presents significant global health challenges, with individuals aged 40+ projected to comprise 22% of the world’s population by 2050 (Bull et al., 2020). Age-related physiological changes—decreased muscle mass, reduced bone density, increased body fat, and declining cardiovascular function—substantially elevate risks of chronic diseases, functional limitations, and premature mortality (Cruz-Jentoft et al., 2019; Papadopoulou, 2020). These changes are particularly pronounced in postmenopausal women due to estrogen decline, which accelerates muscle loss, increases visceral adiposity, and impairs vascular function. Menopausal vasomotor symptoms persist a median of 7.4 years, with symptoms continuing 4.5 years post-menopause, underscoring the long-term health burden in midlife and older women (Avis et al., 2015). Sarcopenia, defined as progressive skeletal muscle mass and strength loss, represents a critical age-related condition associated with increased falls, fractures, disability, and mortality (Cruz-Jentoft et al., 2019; Papadopoulou, 2020). Prevalence varies from 5% to 50% depending on gender, age, diagnostic criteria, and living conditions, with higher rates in nursing homes versus community settings (Papadopoulou, 2020). Given women’s majority in older populations and higher morbidity despite greater longevity—termed the male-female health-survival paradox (Alberts et al., 2014; Oksuzyan et al., 2008)—targeted interventions for aging women are critically needed (Farrelly, 2023).

Cardiorespiratory fitness, assessed through maximal oxygen uptake (VO_2max_), powerfully predicts cardiovascular disease and mortality risk, with each 1-MET increase corresponding to approximately 10%–25% reduction in cardiovascular mortality. While ratio scaling (mL·min^−1^·kg^−1^) is common, models incorporating waist circumference show stronger CVD associations, highlighting body composition’s importance (Salier Eriksson et al., 2021). VO_2max_ reflects integrated cardiovascular, respiratory, and muscular system capacity, making its maintenance critical as age-related decline accelerates. Physical performance measures, including gait speed and functional tests, serve as key sarcopenia severity indicators and predict adverse outcomes (Cruz-Jentoft et al., 2019).

High-intensity interval training (HIIT) has emerged as a time-efficient alternative to moderate-intensity continuous training (MICT), involving repeated vigorous exercise bouts interspersed with recovery, typically performed at ≥80% maximal heart rate or VO_2max_. Adults should accumulate at least 150 min·week^-1^ of moderate-intensity or 75 min·week^-1^ of vigorous-intensity exercise (Bull et al., 2020; Garber et al., 2011). HIIT elicits comparable or superior cardiorespiratory fitness improvements versus MICT with substantially less exercise time, addressing common barriers such as limited time and poor adherence (Wewege et al., 2017). Low-volume sprint interval training (4–6 × 30-s all-out efforts) demonstrates meaningful VO_2max_ increases despite markedly lower training volumes (Gist et al., 2014), involving cellular adaptations including enhanced mitochondrial biogenesis and PGC-1α upregulation. Higher habitual physical activity levels associate with superior vascular function in postmenopausal women (Gliemann et al., 2020). Accumulating sufficient time at or near VO_2max_ through precise interval intensity, duration, and recovery structure is key for maximising aerobic adaptations (Buchheit and Laursen, 2013).

HIIT induces superior vascular improvements (mean flow-mediated dilation: 2.27%) versus MICT (Ramos et al., 2015) and favorable metabolic adaptations, reducing fasting glucose, HbA1c, and improving insulin sensitivity in type 2 diabetes (Jelleyman et al., 2015). Low-volume sprint interval training (∼2.5 h over 2 weeks) produced similar muscle oxidative enzyme, glycogen, and performance improvements as high-volume endurance training (∼10.5 h) (Gibala et al., 2006; Burgomaster et al., 2008). Vigorous-intensity exercise produces greater VO_2max_ improvements than moderate-intensity when volume is controlled (Garber et al., 2011). Program design variables critically influence adaptations; short rest intervals (60 s) induced greater lean mass, strength, and functional gains than extended intervals (4 min) in older men (Villanueva et al., 2015). HIIT improves physical performance and frailty across multiple domains in aged populations (Papadopoulou, 2020).

Several trials demonstrate HIIT improves VO_2max_, body composition, and metabolic parameters in middle-aged and older women. Eight-week interventions increased VO_2max_ and oxygen pulse in obese menopausal women (Dabidi Roshan et al., 2024); 12-week programs reduced blood pressure, improved transcriptomic profiles (Hamelin Morrissette et al., 2022), and decreased body mass, BMI, and fat mass (Jabbour et al., 2025). HIIT proved superior to MICT for reducing abdominal and visceral fat in postmenopausal diabetic women (Maillard et al., 2018). Both HIIT and MICT reduced fat mass (∼2 kg) and waist circumference (∼3 cm), with HIIT requiring 40% less time (Wewege et al., 2017). However, physical performance outcomes remain inconsistent (Tinetti and Kumar, 2010). While HIIT improved handgrip, quadriceps strength, and sit-to-stand performance (Dabidi Roshan et al., 2024), muscle strength rather than mass primarily determines physical performance and predicts outcomes, serving as sarcopenia’s primary diagnostic criterion (Cruz-Jentoft et al., 2019; Newman et al., 2006). Sarcopenia’s multifactorial etiology—hormonal changes, chronic inflammation, oxidative stress, reduced activity, inadequate protein intake (Papadopoulou, 2020)—suggests multifactorial interventions are most effective (Tinetti and Kumar, 2010). Comprehensive programs incorporating cardiorespiratory, resistance, flexibility, and neuromotor training optimize outcomes (Garber et al., 2011), with combined approaches demonstrating 19%–113% strength gains in pre-menopausal women (Murray et al., 2025) and superior improvements when HIIT combined with circuit resistance training (Pashaei et al., 2024).

Despite growing evidence supporting HIIT’s efficacy in older populations, significant gaps persist. First, systematic reviews with meta-analyses specifically evaluating HIIT’s effects on cardiorespiratory fitness and physical function in middle-aged and older women remain limited. Second, optimal HIIT protocols—work-to-rest ratios, session frequency, intervention duration—lack evidence-based consensus. Third, comparative effectiveness versus other modalities requires more rigorous RCT evaluation. Systematic reviews and meta-analyses provide the highest evidence level for clinical decision-making (Thompson and Higgins, 2002). Therefore, this systematic review and meta-analysis, conducted per PRISMA 2020 guidelines (Page et al., 2021), synthesizes current RCT evidence examining HIIT’s effects on cardiorespiratory fitness and physical function in middle-aged and older women.

## 2 Materials and methods

### 2.1 Literature search

The present systematic review and meta-analysis was conducted in accordance with the Preferred Reporting Items for Systematic Reviews and Meta-Analyses (PRISMA) guidelines (Cumpston et al., 2022). The review protocol was prospectively registered in the International Prospective Register of Systematic Reviews (PROSPERO) under the registration number CRD420251272861. A comprehensive literature search was performed in the following electronic databases: PubMed, Web of Science, Scopus. The search covered the period from the inception of each database to [2025.11.30], with no restriction on publication year.

The search strategy was designed to identify randomized controlled trials (RCTs) examining the effects of high-intensity interval training (HIIT) on cardiorespiratory fitness and physical or functional performance in middle-aged and older women. Search terms were developed using combinations of controlled vocabulary (e.g., MeSH terms) and free-text keywords related to the following domains:

1. Population (“middle-aged,” “middle age,” “older adult*,” “elderly,” “aged,” “postmenopausal”);
2. Sex (“female,” “women,” “woman”);
3. Intervention (“high-intensity interval training,” “high intensity interval training,” “HIIT”).

The search strategies were adapted for each database using appropriate syntax and field tags (e.g., title, abstract, and keywords). The detailed search strategies for all databases are provided in Supplementary Material.

### 2.2 Literature search inclusion and exclusion criteria

**FIGURE 1:**
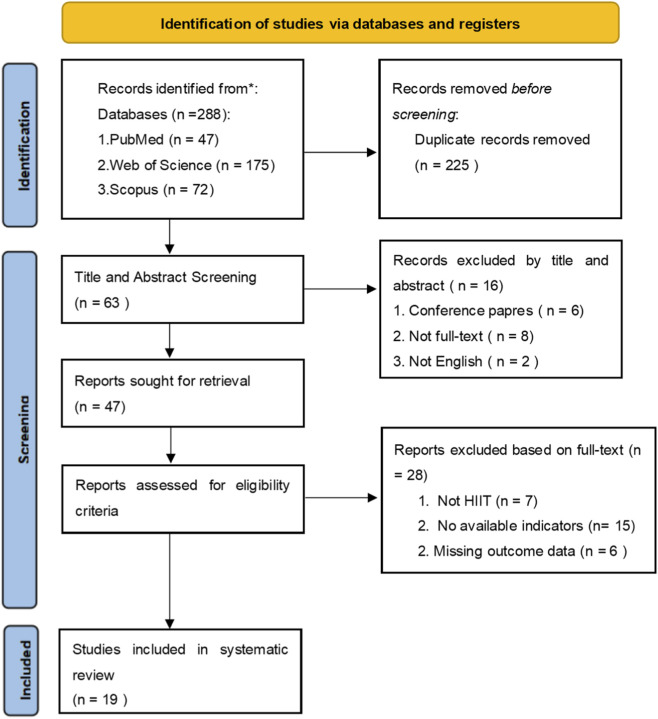
PRISMA.

The retrieved literature was imported into Zotero software for management. Two researchers independently screened the literature by reviewing titles, abstracts, and full texts, removing duplicate and irrelevant studies, and extracting eligible data. Any disagreements during the screening process were resolved through discussion, and when necessary, consultation with a third reviewer. The literature screening process is illustrated in Figure 1.

The target population of this review consisted of middle-aged and older women, including peri- and postmenopausal women. Middle-aged was operationally defined as women aged 40 years and older, consistent with exercise physiology research definitions. Although participant ages ranged from 44 to 81 years across included studies, this broad inclusion was physiologically justified. From age 40 onward, women experience progressive hormonal changes, declining VO_2max_ (approximately 10% per decade), muscle mass loss, and increasing cardiovascular risk. These age-related physiological changes, rather than chronological age alone, represent the therapeutic target for HIIT interventions. Subgroup analyses by age (<65 vs. ≥ 65 years) were conducted where feasible to explore potential heterogeneity. There were no restrictions on country, ethnicity, or baseline health status, except for professional or competitive athletes. Only studies involving human participants were included.

The intervention was high-intensity interval training (HIIT), defined as repeated bouts of high-intensity exercise interspersed with recovery periods. The control group included non-exercise control, usual care, moderate-intensity continuous training, or other low-to moderate-intensity exercise interventions.

Only randomized controlled trials (RCTs) published in English or Chinese were eligible for inclusion. Studies were required to report at least one outcome related to cardiorespiratory fitness or physical/functional performance.

The outcome measures included indicators of cardiorespiratory fitness, such as maximal or peak oxygen uptake (VO_2max_/VO_2peak_), estimated VO_2max_; and indicators of physical or functional performance, such as 6-min walk test (6MWT), Timed Up and Go (TUG), sit-to-stand or chair-stand tests, and time to exhaustion, gait speed, handgrip strength, and one-repetition maximum.

### 2.3 Data extraction

The data extraction included the following information: basic characteristics of the included studies (first author’s name, publication year, and country), participant characteristics (mean age, sex, sample size, and health status), intervention characteristics (exercise modality, intensity prescription, interval structure, frequency, and intervention duration), comparator details, outcome measures related to cardiorespiratory fitness and physical or functional performance, and key items for risk of bias assessment.

For the quantitative synthesis, means and standard deviations (SDs) of outcome measures at baseline and post-intervention were extracted for both intervention and control groups. When change-from-baseline values were directly reported, these data were preferentially extracted.

If outcome data were reported in alternative formats (e.g., standard errors, confidence intervals, or medians and interquartile ranges), they were converted to means and SDs using methods described in the Cochrane Handbook (Higgins et al., 2011) and by Wan et al. (Wan et al., 2014) when possible.

In cases of missing or unclear data, attempts were made to contact the corresponding authors to obtain the required information. Studies were excluded from the quantitative analysis if the necessary data could not be retrieved or reliably estimated. The detailed characteristics of the included studies are summarized in Supplementary Table S1.

### 2.4 Risk of bias assessment and summary of evidence

RCTs were analyzed using the Cochrane Risk of Bias Tool 2.0 (Purgato et al., 2010). There are three levels: low risk, high risk, and uncertain. Two researchers used ReviewManager 5.4.1 software to rigorously evaluate five aspects of randomized allocation methods, allocation concealment of randomized methods, blinding of research subjects and interveners, blinding of outcome evaluators, integrity of outcome data, possibility of selective reporting, and other sources of bias. The risk of biased judgment in each domain was interpreted as low risk, moderate risk, severe risk, borderline risk, or no information. Two reviewers independently assessed the risk of bias, and any disagreements were resolved through a third party. In addition, the risk of publication bias was assessed using funnel plots when the meta-analysis included ≥5 studies.

### 2.5 Statistical analysis

All statistical analyses were conducted using Review Manager (RevMan) version 5.4.1. Meta-analyses were performed when at least two studies reported comparable outcomes. Outcomes related to cardiorespiratory fitness (as VO_2max_ and VO_2peak_) and physical or functional performance (as 6-min walk test, Timed Up and Go, sit-to-stand performance, gait speed, muscle strength, and flexibility) were analyzed separately.

For continuous outcomes measured using the same unit, mean difference (MD) with 95% confidence intervals (CIs) was calculated. When outcomes were assessed using different scales or measurement methods, standardized mean difference (SMD) with 95% CIs was used. Given the expected clinical and methodological heterogeneity across studies (as differences in training protocols, intervention duration, participant characteristics, and outcome assessment methods), a random-effects model was applied for all meta-analyses. Statistical heterogeneity was assessed using the I^2^ statistic and the Chi-square test, with I^2^ values of approximately 25%, 50%, and 75% indicating low, moderate, and high heterogeneity, respectively.

Prespecified subgroup analyses were conducted to explore potential sources of heterogeneity according to age (<65 vs. ≥ 65 years), population characteristics (obese vs. non-obese), intervention duration, and training frequency. Sensitivity analyses were performed by sequentially excluding individual studies to assess the robustness of the pooled estimates. When at least five studies were included in a meta-analysis, publication bias was assessed visually using funnel plots.

The overall certainty of evidence for primary outcomes was evaluated using the GRADE approach (Prasad, 2024), considering risk of bias, inconsistency, indirectness, imprecision, and publication bias. A two-sided P value <0.05 was considered statistically significant for all analyses. Meta-regression was not performed due to insufficient studies (<10) for each outcome (Thompson and Higgins, 2002).

## 3 Results

### 3.1 Study selection

Figure 1 presents the flowchart of the literature screening process. A total of 288 relevant records were identified through searches of three databases (PubMed:47 articles, Web of Science: 175 articles, Scopus: 72 articles). After removing 225 duplicate publications, 63 articles proceeded to the screening process. During the title and abstract screening phase, 16 articles were excluded. During the full-text screening phase, 28 articles were excluded. Finally, 19 studies were included in the meta-analysis as Table 1 (Gliemann et al., 2020; Teixeira Do Amaral et al., 2024; Ballesta-García et al., 2019; Chen et al., 2024; Coswig et al., 2020; Dupuit et al., 2020a; Dupuit et al., 2022; Dupuit et al., 2020b; Henke et al., 2018; Kazemi et al., 2023; Klonizakis et al., 2014; Marcotte-Chénard et al., 2021; Noorbakhsh and Dabidi Roshan, 2023; Norling et al., 2024; Pan et al., 2025; Steckling et al., 2019; Twerenbold et al., 2023; Yu et al., 2025; Zanini et al., 2025).

### 3.2 Characteristics of included studies

The included studies involved a total of 646 middle-aged and older women, with mean ages ranging from approximately 44–81 years. Across the studies, participants included healthy women as well as those who were overweight or obese, postmenopausal, or with cardiometabolic risk factors. The intervention group consisted of participants receiving high-intensity interval training (HIIT), while control groups included non-exercise controls, moderate-intensity continuous training, resistance training, or combined exercise interventions.

The training frequency ranged from 2 to 4 sessions per week, and the intervention duration varied from 2 weeks to 9 months. HIIT protocols differed in exercise modality, including cycling, treadmill walking or running, Nordic walking, and sport-based interval training, with exercise intensity generally prescribed using heart rate, oxygen uptake, power output, or perceived exertion. Outcome measures primarily assessed cardiorespiratory fitness, such as VO_2max_ or VO_2peak_, and physical or functional performance, including the 6-min walk test, Timed Up and Go, sit-to-stand performance, gait speed, muscle strength, and flexibility.

Most studies reported supervised exercise interventions; however, the level of detail regarding supervision varied across trials. Participant withdrawals were reported in several studies, commonly due to personal reasons or non-exercise-related factors.

**TABLE 1:** Characteristics of included studies.

Study	Age (mean)	Physical condition	Cycle	Frequency	Control group	Intervention intensity	Intervention program	Outcomes
Teixeira Do Amaral et al. (2024)	Older women	Low-income older women	9 months	2 sessions/week	MICT + RT	HIIT: high intensity; MICT: moderate intensity	Community-based HIIT + RT vs. MICT + RT vs. RT	VO_2max_/VO_2peak_
Ballesta-garcía et al. (2019)	67.8 years	Functionally independent middle-aged and older women	18 weeks	2 sessions/week	Non-exercise control	HIICT: RPE 14–18; MICT: RPE 9–14	High-intensity vs. moderate-intensity circuit training	6-min walk test (6MWT) Timed up and go (TUG); 30-s chair stand
Chen et al. (2024)	44.5 years	Obese middle-aged women	8 weeks	3 sessions/week	Placebo/probiotic	HIIT: 85%–90% vVO_2max_	HIIT with or without probiotic supplementation	VO_2max_; time to exhaustion (TTE); running economy (RE)
Coswig et al. (2020)	80.8 years	Institutionalized elderly women	8 weeks (+detraining)	2 sessions/week	MIIT/MICT	HIIT: 85%–95% HRmax	HIIT vs. MIIT vs. MICT treadmill training	6-min walk test (6MWT) 30-s chair stand; gait speed (10-m walk)
Dupuit et al.(2020b)	∼61 years	Overweight postmenopausal women	12 weeks	3 sessions/week	MICT	HIIT: ∼85% HRpeak	HIIT vs. MICT	VO_2max_; peak power output (PPO)
Dupuit et al. (2020a)	62.4 years	Overweight/obese postmenopausal women	12 weeks	3 sessions/week	MICT	HIIT: ∼85% HRpeak	HIIT vs. MICT vs. HIIT + RT	VO_2max_; peak power output (PPO)
Dupuit et al. (2022)	∼63 years	Postmenopausal women with cardiometabolic risk	12 weeks	3 sessions/week	MICT	HIIT: high intensity (HR-based)	HIIT vs. MICT	VO_2max_/VO_2peak_
Gliemann et al. (2020)	62.2 years	Late postmenopausal women	10 weeks	2 sessions/week	No-exercise control	Intermittent high intensity (HR >85% HRmax)	Floorball-based HIIT (small-sided games)	VO_2max_/VO_2peak_
Henke et al. (2018)	58.3 years	Sedentary obese postmenopausal women	4 weeks	2 sessions/week	No-exercise control	HIIT: 85%–90% HRmax	Cycle-ergometer HIIT	VO_2peak_; time to exhaustion
Kazemi et al. (2023)	∼55 years	Postmenopausal women with metabolic syndrome	8 weeks	3 sessions/week	Non-exercise control	HIIT: 80%–90% HRmax; RT: 75%–80% 1RM	HIIT vs. RT vs. control	VO_2peak_; 6-min walk test (6MWT) 1-RM strength (upper and lower body)
Klonizakis et al. (2014)	64 years	Postmenopausal women, physically inactive	2 weeks	3×/week (6 sessions total)	Moderate-intensity continuous training (CT)	HIIT: 100% peak power output; CT: 65% peak power	Cycling HIIT: 10 × 1-min at 100% PPO with 1-min active recovery vs. 40-min continuous cycling	Peak oxygen uptake (VO_2peak_)
Marcotte-Chénard et al. (2021)	∼68 years	Older women with type 2 diabetes, inactive	12 weeks	3×/week	MICT	HIIT: ∼90% HRR; MICT: ∼60% HRR	Walking HIIT on treadmill: 6 × 1-min at 90% HRR with 2-min recovery; MICT continuous walking	VO_2peak_ Muscular endurance (sit-to-stand test)
Noorbakhsh and Dabidi Roshan (2023)	∼50 years	Overweight/pre-obese elderly women	8 weeks	2×/week	Placebo	Tabata-HIIT: 80%–90% HRmax	Tabata HIIT (20 s work/20 s rest, progressive sets); some arms combined with nanocurcumin supplementation	Lower-body power; functional performance tests
Norling et al. (2024)	∼66 years	Sedentary older women, cognitively healthy	8 weeks	4×/week	No non-exercise control	70%–90% HRmax (progressive)	Cycling HIIT: 10 × 1-min intervals with 1–2-min active recovery, supervised	VO_2max_/VO_2peak_
Pan et al. (2025)	68.9 years	Postmenopausal women without sarcopenia	12 weeks	3×/week	Strength training group + non-exercise control	HIIT-NW: 75%–80% HRmax	High-intensity interval Nordic walking: 60-s high-intensity bouts with 60-s rest; compared with traditional strength training	Cardiorespiratory endurance (estimated VO_2max_) Lower-limb strength; functional walking capacity
Steckling et al. (2019)	∼67 years	Postmenopausal women with metabolic syndrome	12 weeks	3×/week	MICT	HIIT: 85%–95% HRmax; MICT: 60%–70% HRmax	Treadmill-based HIIT (4 × 4-min intervals) vs. continuous aerobic training	VO_2peak_ Time-to-exhaustion
Twerenbold et al. (2023)	∼63 years	Overweight/obese older women with cardiovascular risk	12 weeks	3×/week	Non-exercise control	HIIT: ≥90% HRpeak	Supervised cycling HIIT with short high-intensity bouts and active recovery	VO_2peak_
Yu et al. (2025)	47.0 years	Obese middle-aged women with prehypertension	6 weeks	3×/week	RT + MICT	HIIT: 85%–95% HRpeak; RT: OMNI 6–7	Concurrent training: resistance training + treadmill HIIT (4 × 4-min at 85%–95% HRpeak) vs. RT + MICT	VO_2peak_ (GXT, Bruce protocol) Grip strength; sit-ups; sit-and-reach
Zanini et al. (2025)	71–74 years	Older women under socioeconomic vulnerability	6 months	2×/week	MICT + RT	HIIT: RPE 15–17; MICT: RPE 11–13	Community-based program: walking/jogging HIIT + resistance training vs. MICT + RT and RT alone	6-min walk test (6MWT) Five-time sit-to-stand; timed up and go; handgrip strength

HIIT, high-intensity interval training; MICT, moderate-intensity continuous training; RT, resistance training; VO_2max_, maximal oxygen uptake; VO_2peak_, peak oxygen uptake; HRmax, maximal heart rate; HRR, heart rate reserve; RPE, rating of perceived exertion; 6MWT, 6-min walk test; TUG, Timed Up and Go test. Detailed intervention protocols are provided in Supplementary Table S1.

### 3.3 Quality assessment of included studies

**FIGURE 2:**
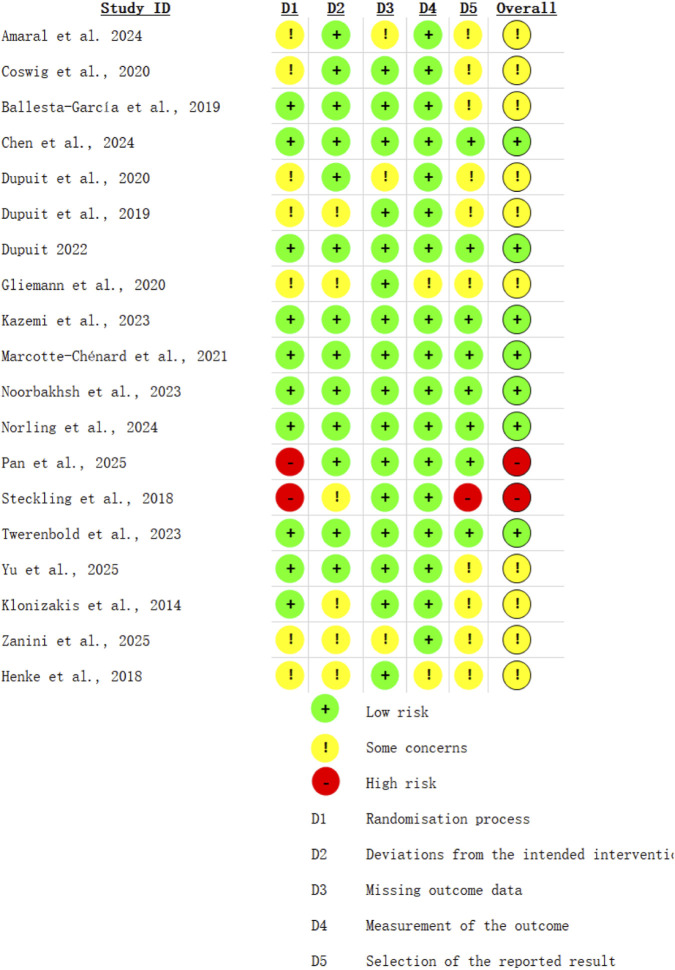
Risk of bias assessment.

**FIGURE 3:**
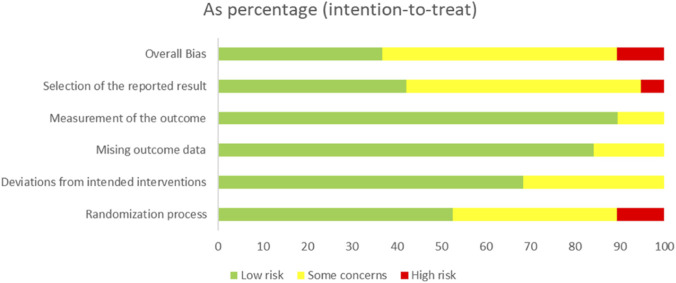
Risk of bias summary.

Risk of bias assessment using Cochrane RoB 2 showed variable quality across 19 RCTs (Figures 2, 3). Most studies had low risk for randomization (63%), missing data (74%), and selective reporting (84%). Overall, 42% had low risk of bias, 53% raised some concerns, and 5% had high risk.

### 3.4 Meta-analysis

#### 3.4.1 Meta-analysis of physical performance

**FIGURE 4:**
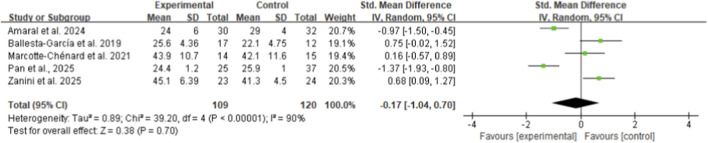
Meta-analysis of skeletal muscle strength performance.

**FIGURE 5:**
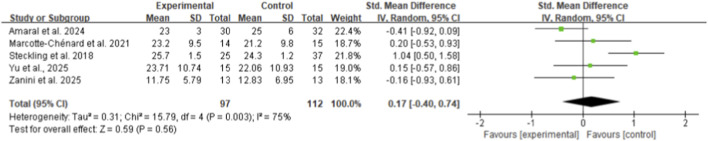
Meta-analysis of flexibility performance.

From five included studies involving middle-aged and older women, HIIT showed no significant effect on skeletal muscle strength performance compared to control groups, with a standardized mean difference (SMD) of −0.17 (95% CI: −1.04 to 0.70, p = 0.70, I^2^ = 89%) (Figure 4) (Teixeira Do Amaral et al., 2024; Ballesta-García et al., 2019; Marcotte-Chénard et al., 2021; Pan et al., 2025; Zanini et al., 2025). Similarly, flexibility performance demonstrated no significant improvement following HIIT intervention, with an SMD of 0.17 (95% CI: −0.40 to 0.74, p = 0.56, I^2^ = 75%) based on five studies (Figure 5) (Teixeira Do Amaral et al., 2024; Marcotte-Chénard et al., 2021; Steckling et al., 2019; Yu et al., 2025; Zanini et al., 2025).

**FIGURE 6:**
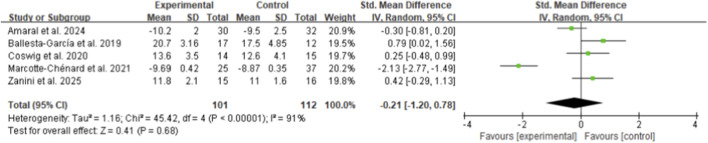
Meta-analysis of standing ability.

**FIGURE 7:**
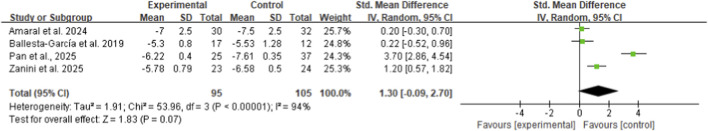
Meta-analysis of standing up and walking ability.

**FIGURE 8:**
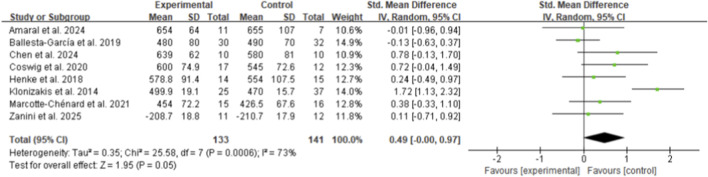
Meta-analysis of walking ability.

For functional mobility outcomes, five studies examining standing ability (sit-to-stand performance) revealed no significant differences between HIIT and control groups (SMD: −0.21, 95% CI: −1.20 to 0.78, p = 0.68, I^2^ = 91%) (Figure 6) (Teixeira Do Amaral et al., 2024; Ballesta-García et al., 2019; Coswig et al., 2020; Marcotte-Chénard et al., 2021; Zanini et al., 2025). Four studies assessing Timed Up and Go (TUG) performance showed a large but non-significant effect favoring HIIT (SMD 1.30, 95% CI: −0.09 to 2.70, p = 0.07, I^2^ = 94%) (Figure 7) (Teixeira Do Amaral et al., 2024; Ballesta-García et al., 2019; Pan et al., 2025; Zanini et al., 2025). Walking ability, measured by the 6-min walk test across eight studies, demonstrated a borderline significant improvement with HIIT intervention (SMD 0.49, 95% CI: 0.00 to 0.97, p = 0.05, I^2^ = 73%) (Figure 8) (Teixeira Do Amaral et al., 2024; Ballesta-García et al., 2019; Chen et al., 2024; Coswig et al., 2020; Henke et al., 2018; Klonizakis et al., 2014; Marcotte-Chénard et al., 2021; Zanini et al., 2025).

#### 3.4.2 Meta-analysis of cardiopulmonary function

**FIGURE 9:**
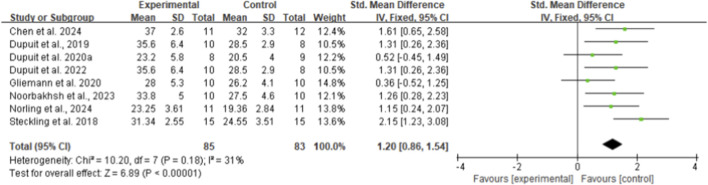
Meta-analysis of VO_2max_.

**FIGURE 10:**
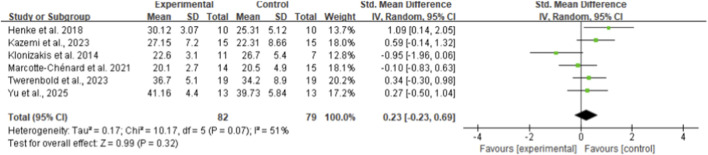
Meta-analysis of VO_2Peak_.

From eight included studies, HIIT was found significantly more effective in improving maximal oxygen uptake (VO_2max_) compared to control interventions, with an SMD of 1.20 (95% CI: 0.86 to 1.54, p < 0.01, I^2^ = 31%) (Figure 9) (Gliemann et al., 2020; Chen et al., 2024; Dupuit et al., 2020a; Dupuit et al., 2022; Dupuit et al., 2020b; Noorbakhsh and Dabidi Roshan, 2023; Norling et al., 2024; Steckling et al., 2019). In contrast, six studies examining peak oxygen uptake (VO_2peak_) showed no significant difference between HIIT and control groups (SMD 0.23, 95% CI: −0.23 to 0.69, p = 0.32, I^2^ = 51%) (Figure 10) (Henke et al., 2018; Kazemi et al., 2023; Klonizakis et al., 2014; Marcotte-Chénard et al., 2021; Twerenbold et al., 2023; Yu et al., 2025).

### 3.5 Subgroup and sensitivity analysis

Subgroup analyses were performed for walking ability as Table 2, VO_2peak_, and VO_2max_ as these outcomes included sufficient studies to allow meaningful stratified comparisons. For walking ability, participants ≥65 years showed a borderline significant improvement (5 studies, SMD 0.65, 95% CI: 0.00 to 1.30, p = 0.05, I^2^ = 74%). Training frequency analysis revealed a significant effect with 2 sessions per week (4 studies, SMD 0.93, 95% CI: 0.29 to 1.58, p = 0.005, I^2^ = 68%). Other subgroups showed no significant effects. For VO_2peak_, only participants <64 years showed significant improvements (4 studies, SMD 0.50, 95% CI: 0.13 to 0.88, p = 0.009, I^2^ = 0%).

For VO_2max_, significant improvements were observed across all subgroups: age (<65 years: 5 studies, SMD 1.19, 95% CI: 0.51 to 1.87, p = 0.0006, I^2^ = 60%; ≥65 years: 3 studies, SMD 1.23, 95% CI: 0.67 to 1.80, p = 0.0001, I^2^ = 0%); population (non-obese: 2 studies, SMD 0.74, 95% CI: 0.11 to 1.38, p = 0.02, I^2^ = 32%; obese: 6 studies, SMD 1.38, 95% CI: 0.98 to 1.78, p < 0.0001, I^2^ = 17%); and intervention duration (≤10 weeks: 4 studies, SMD 1.06, 95% CI: 0.60 to 1.53, p < 0.0001, I^2^ = 22%; ≥12 weeks: 4 studies, SMD 1.35, 95% CI: 0.85 to 1.84, p < 0.0001, I^2^ = 47%).

**TABLE 2:** Subgroup analysis of main indicators.

Walking ability					
Study or subgroup	n	SMD	95% CI	I^2^(%)	P
Age					
<65 years	3	0.16	[−0.36, 0.64]	32	0.58
>65 years	5	0.65	[0.00, 1.30]	74	0.05
Population (cycle)					
Non-obese (≤2 months)	4	0.36	[−0.02, 0.75]	0	0.07
Obese (>2 months)	4	0.62	[−0.33, 1.57]	87	0.2
Frequency					
2 times/weeks	4	0.93	[0.29, 1.58]	68	0.005
3 times/weeks	4	0.01	[−0.34, 0.35]	0	0.97

VO_2_Peak					
Study or subgroup	n	SMD	95% CI	I^2^(%)	P
Age					
<64 years	4	0.5	[0.13, 0.88]	0	0.009
≥65 years	2	-0.39	[-0.98, 0.20]	44	0.2
Population					
Non-obese	4	0.05	[-0.52, 0.62]	55	0.87
Obese	2	0.63	[-0.17, 1.43]	42	0.12
Cycle					
≤6 weeks	3	0.15	[-0.92, 1.22]	76	0.78
>6 weeks	3	0.28	[-0.12, 0.68]	0	0.17

VO_2_Max					
Study or subgroup	n	SMD	95% CI	I^2^(%)	P
Age					
<65 years	5	1.19	[0.51, 1.87]	60	0.0006
≥65 years	3	1.23	[0.67, 1.80]	0	0.0001
Population					
Non-obese	2	0.74	[0.11, 1.38]	32	0.02
Obese	6	1.38	[0.98, 1.78]	17	<0.0001
Cycle					
≤10 weeks	4	1.06	[0.60, 1.53]	22	<0.0001
≥12 weeks	4	1.35	[0.85, 1.84]	47	<0.0001

SMD, standardized mean difference; CI, confidence interval; I^2^, heterogeneity statistic. Subgroups defined a priori based on age (<65 vs. ≥ 65 years), body mass status (obese vs. non-obese), intervention duration (≤10 weeks vs. ≥ 12 weeks), and training frequency (sessions per week). P-values for subgroup differences not reported due to limited number of studies in some subgroups.

Sensitivity analyses indicated that the pooled effects for most outcomes were robust to the exclusion of individual studies, with the exception of one outcome that demonstrated substantial dependence on a single influential study. Overall, these findings suggest that the main conclusions of the meta-analysis are generally stable, although results for outcomes with high heterogeneity and limited numbers of studies should be interpreted with caution (As Supplementary Figure S1).

### 3.6 Publication bias

**FIGURE 11:**
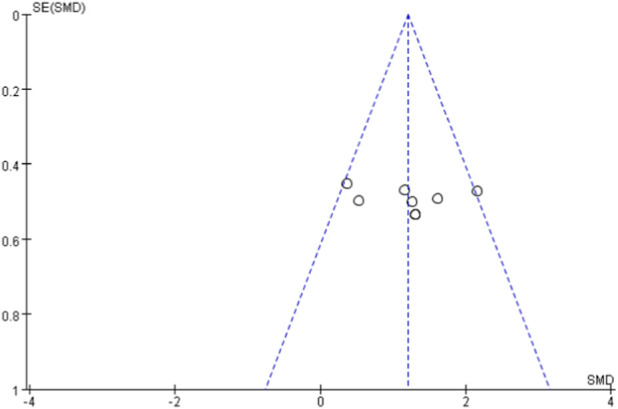
Funnel diagram of VO_2max_.

**FIGURE 12:**
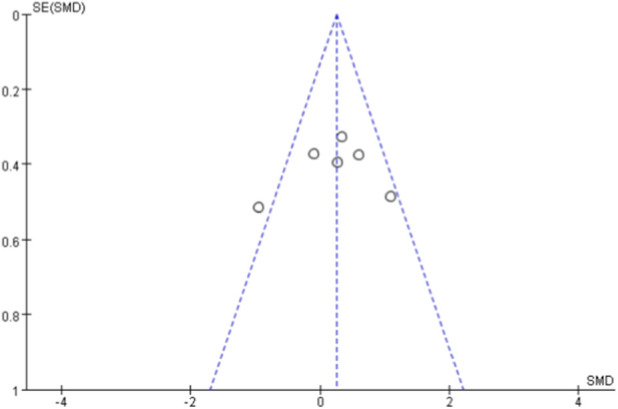
Funnel diagram of VO_2peak_.

**FIGURE 13:**
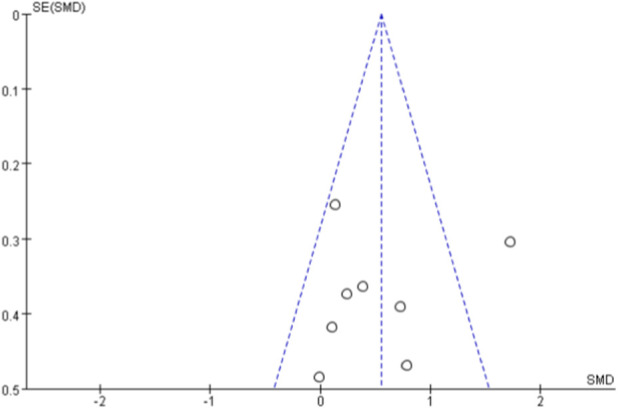
Funnel diagram of Walking ability.

Due to the limited number of studies included in each meta-analysis (<10 studies for all outcomes), statistical tests for asymmetry (e.g., Egger’s test) were not performed. Publication bias was assessed using funnel plots for outcomes with ≥5 studies (Sterne et al., 2011). Funnel plots for VO_2max_ (Figure 11) and VO_2peak_ (Figure 12) showed relatively symmetrical distribution of studies. The funnel plot for walking ability (Figure 13) showed some asymmetry with fewer studies on the left side, suggesting potential publication bias for this outcome.

### 3.7 GRADE recommendations

The strength of evidence according to GRADE criteria generated by the GRADEpro website (https://www.gradepro.org/) is listed on Table 3.

**TABLE 3:** GRADE recommendations.

Certainty assessment						Summary of findings			Overall certainty of evidence
Participants (Studies)	Risk of bias	Inconsistency	Indirectness	Imprecision	Publication bias	Study event rates (%)		Anticipated absolute effects	
Follow-up						With placebo	With HIIT	Risk difference with HIIT	
Muscle strength performance									
229	Not serious	Very serious	Not serious	Serious	None	120	109	SMD −0.17 lower (−1.04 lower to 0.7 higher)	⨁
(5 RCTs)									Very low
Flexibility performance									
209	Not serious	Serious	Not serious	Serious	None	112	97	SMD 0.17 higher (−0.4 lower to 0.74 higher)	⨁⨁
(5 RCTs)									Low
Standing ability									
213	Not serious	Very serious	Not serious	Serious	None	112	101	SMD 0.21 lower (1.2 lower to 0.78 higher)	⨁
(5 RCTs)									Very low
Standing up and walking ability									
200	Not serious	Very serious	Not serious	Serious	None	105	95	SMD 0.97 higher (0.65 higher to 1.28 higher)	⨁
(4 RCTs)									Very low
Walking ability									
274	Not serious	Serious	Not serious	Serious	Publication bias strongly suspected	141	133	SMD 0.49 higher (0.0 higher to 0.97 higher)	⨁
(8 RCTs)									Very low
VO_2Peak_									
161	Not serious	Serious	Not serious	Serious	None	79	82	SMD 0.23 higher (−0.23 lower to 0.69 higher)	⨁⨁
(6 RCTs)									Low
VO_2max_									
168	Not serious	Not serious	Not serious	Not serious	None	83	85	SMD 1.2 higher (0.86 higher to 1.54 higher)	⨁⨁⨁⨁
(8 RCTs)									High

GRADE, grading of recommendations assessment, Development and Evaluation; SMD, standardized mean difference; CI, confidence interval; Certainty ratings: High (⊕⊕⊕⊕) = further research very unlikely to change confidence in estimate; Moderate (⊕⊕⊕○) = further research likely to have important impact; Low (⊕⊕○○) = further research very likely to have important impact; Very low (⊕○○○) = very uncertain about the estimate; Certainty downgraded for: risk of bias, inconsistency (I^2^), indirectness, imprecision, publication bias.

For cardiorespiratory fitness outcomes, VO_2max_ was rated as high-quality evidence, showing a significant improvement with HIIT (SMD 1.20, 95% CI: 0.86–1.54) and low heterogeneity (I^2^ = 31%). VO_2peak_ was rated as low-quality evidence, downgraded by two levels due to moderate inconsistency (I^2^ = 51%) and imprecision (wide confidence interval crossing the line of no effect).

For physical performance outcomes, all indicators demonstrated very low to low-quality evidence. Walking ability was rated as very low-quality evidence, downgraded by three levels for serious inconsistency (I^2^ = 73%), imprecision (confidence interval touching null), and suspected publication bias (asymmetric funnel plot). Timed Up and Go, sit-to-stand performance, and muscle strength were all rated as very low-quality evidence, primarily downgraded for very serious inconsistency (I^2^ = 89%–94%) and imprecision. Flexibility was rated as low-quality evidence, downgraded by two levels for serious inconsistency (I^2^ = 75%) and imprecision.

## 4 Discussion

This systematic review evaluated HIIT’s effects on cardiorespiratory fitness and physical function in middle-aged and older women. HIIT significantly improved VO_2max_ with high-certainty evidence but showed limited effects on physical function, suggesting HIIT alone is insufficient for comprehensive functional improvement. However, the robust cardiorespiratory gains highlight HIIT as a valuable component within multimodal exercise programs (Herbert et al., 2017).

### 4.1 HIIT effects on cardiorespiratory fitness

Sex-specific considerations. Postmenopausal estrogen decline accelerates cardiovascular aging and impairs muscle protein synthesis, potentially modifying HIIT responses compared to men. Previous mixed-sex meta-analyses may mask these differences. Our women-focused approach addresses this gap, though direct sex comparisons await future research.

Meta-analysis showed HIIT significantly improved VO_2max_ (SMD 1.20, 95% CI: 0.86–1.54, I^2^ = 31%), as detailed in Results. These cardiorespiratory gains are clinically meaningful given the well-established association between fitness level and reduced mortality risk in women (Womack et al., 2003). HIIT produced greater VO_2max_ improvements than moderate-intensity training across diverse populations (Milanović et al., 2015).

Even ultra-short interventions demonstrate rapid adaptations. A 2-week low-volume HIIT protocol (six sessions, 10 × 1-min intervals at 100% peak power) increased VO_2peak_ by 2.2 mL·kg^−1^·min^−1^ (11%, P = 0.01) in postmenopausal women with half the training volume (558 vs. 1,237 kJ) and time (2.5 vs. 5 h) versus moderate-intensity training (Klonizakis et al., 2014). An 8-week Tabata-style HIIT protocol (twice weekly at 80%–90% HRmax) significantly improved VO_2max_ and oxygen pulse in overweight elderly women (Noorbakhsh and Dabidi Roshan, 2023). In sedentary older women (60–75 years), 8-week HIIT increased VO_2max_ by 20% (19.36–23.25 mL·kg^−1^·min^−1^, p < 0.001) with 97% adherence, alongside significant cognitive improvements (Norling et al., 2024).

In older women with type 2 diabetes, 12-week low-volume walking HIIT (75 min/week: 6 × 1-min at 90% HRR) produced similar VO_2peak_ improvements (+7.6%) as moderate-intensity training (150 min/week, +7.0%) despite 50% less training time, with superior improvements in 6-min walk distance (HIIT: +98 ± 56 m vs. MICT: +28 ± 70 m, P = 0.01) and grip strength (HIIT: +3.8 ± 5.5 kg vs. MICT: +0.1 ± 1.9 kg, P = 0.02) (Marcotte-Chénard et al., 2021). Subgroup analyses showed consistent VO_2max_ benefits across ages when sessions met vigorous-intensity guidelines (≥75 min·week^−1^) (Dupuit et al., 2020a; Dupuit et al., 2022).

High compliance rates (>85%) suggest HIIT feasibility in this population, though some women report higher perceived exertion during intervals versus continuous exercise (Jung et al., 2014).

### 4.2 HIIT effects on physical function and muscle strength

HIIT did not significantly improve muscle strength, with only borderline effects on walking ability. Low muscle strength is sarcopenia’s main diagnostic criterion and predicts disability, falls, and mortality (Cruz-Jentoft et al., 2019; Newman et al., 2006). Age-related muscle loss involves neuromuscular, hormonal, and inflammatory changes not fully addressed by HIIT alone.

A 12-week trial comparing HIIT-based Nordic walking with strength training in postmenopausal women (60–79 years) showed both interventions prevented sarcopenia: HIIT Nordic walking improved lower limb strength (chair stand, knee flexor strength, timed up-and-go) and skeletal muscle index, while strength training enhanced upper limb strength (hand grip, arm curl) and reduced body fat (Pan et al., 2025). Notably, HIIT interventions appear to predominantly benefit lower-body strength, with less consistent effects on upper-body strength measures such as handgrip strength (Pan et al., 2025), suggesting potential site-specific adaptations to this training modality.

High-intensity and moderate-intensity circuit training produced similar lower-limb strength and balance improvements, with additional upper-limb gains in the high-intensity group (Ballesta-García et al., 2019).

In elderly nursing home residents, 8-week HIIT (4 × 4-min at 85%–95% HRmax) improved chair stand and 6-min walk tests more than moderate-intensity training, with sustained benefits after 2–4 weeks detraining while other groups declined below baseline (Coswig et al., 2020). Walking performance improvements are clinically relevant for independence and survival (Studenski et al., 2011), but larger trials are needed to determine if HIIT alone reliably improves gait speed without targeted strength and balance training (Garber et al., 2011).

### 4.3 Clinical implications and combined approaches

Beyond cardiorespiratory benefits, HIIT favorably modulates metabolic and inflammatory profiles. In postmenopausal women with metabolic syndrome, 12-week HIIT (4 × 4-min at 90% HRmax, 3×/week) improved VO_2max_ (+27.7%), body composition, glucose control, and blood pressure while reducing inflammatory cytokines (IL-6, IL-18, TNF-α, IFN-γ) and increasing anti-inflammatory IL-10, alongside beneficial adipokine shifts (increased adiponectin; decreased resistin, leptin, ghrelin) (Steckling et al., 2019). Even 8-week HIIT demonstrated microvascular endothelial improvements in hypertensive patients, with significant increases in retinal arteriolar flicker-induced dilation independent of blood pressure changes (Twerenbold et al., 2023).

Combined interventions may amplify benefits. Tabata-HIIT plus nanocurcumin supplementation produced superior improvements in body composition, VO_2max_, and inflammasome suppression versus exercise alone (Noorbakhsh and Dabidi Roshan, 2023). In obese middle-aged women with prehypertension, 6-week concurrent training combining resistance exercise with HIIT showed superior blood lipid improvements (LDL-C, HDL-C, LDL-C/HDL-C ratio) compared to resistance plus moderate-intensity training (Yu et al., 2025).

In socioeconomically vulnerable older women, 6-month community-based concurrent training (HIIT + RT, MICT + RT, or RT alone, twice weekly) yielded comparable improvements in lower limb strength, mobility, aerobic performance, and mood profile regardless of exercise intensity, demonstrating accessible community programs can improve physical function and mental health (Zanini et al., 2025).

Implementation strategies should consider individual preferences, time constraints, facility access, and supervision requirements to maximize adherence. Optimal prescription parameters—session frequency, interval duration, intensity targets, progression schemes—remain to be fully elucidated for diverse subpopulations.

### 4.4 Heterogeneity and sources of variation

Substantial heterogeneity was observed for muscle strength (I^2^ = 89%), sit-to-stand performance (I^2^ = 91%), Timed Up and Go (I^2^ = 94%), and flexibility (I^2^ = 75%). High I^2^ values (>75%) indicate considerable inconsistency that may reflect true differences rather than sampling variation alone (Higgins et al., 2003).

Methodological sources. In our meta-analysis of muscle strength outcomes, handgrip dynamometry was the primary assessment method due to its standardization and prevalence across included studies (Dabidi Roshan et al., 2024). While other strength measures (e.g., one-repetition maximum testing, isokinetic dynamometry) capture distinct neuromuscular aspects (Roberts et al., 2011), insufficient data precluded their inclusion in the pooled analysis.

Sit-to-stand tests employed different protocols (30-s chair stand versus five-repetition timed tests), assessing different capacities (muscular endurance versus power).

HIIT protocols differed substantially in modality. Cycle ergometry-based HIIT (Dupuit et al., 2020a; Dupuit et al., 2022; Klonizakis et al., 2014) may elicit different neuromuscular adaptations than weight-bearing modalities such as treadmill walking (Marcotte-Chénard et al., 2021) or Nordic walking (Pan et al., 2025), as non-weight-bearing exercise reduces mechanical loading. Additionally, intensity prescription methods, interval structure (work-to-rest ratios: 1:1 to 1:4), and session duration varied, influencing adaptation magnitude and specificity (Buchheit and Laursen, 2013).

#### 4.4.1 Participant-related sources

Baseline characteristics varied widely: age (44–81 years), health status (healthy postmenopausal women (Dupuit et al., 2022; Marcotte-Chénard et al., 2021; Steckling et al., 2019), obesity, metabolic syndrome, type 2 diabetes, hypertension), and functional capacity. Physiological responses may differ substantially between healthy middle-aged women and older adults with comorbidities. Metabolic or cardiovascular conditions may modulate training adaptations through altered inflammatory, hormonal, or vascular responses.

#### 4.4.2 Intervention-related factors

Intervention duration (2 weeks–9 months) and training frequency (2–4 sessions/week) varied substantially. Subgroup analyses revealed some stratifications reduced heterogeneity (e.g., VO_2max_: I^2^ = 0% in participants ≥65 years), suggesting age-specific responses. However, for most physical function outcomes, subgroup analyses did not fully resolve heterogeneity, consistent with recognized limitations when study numbers are small (Thompson and Higgins, 2002). Unmeasured factors—supervision intensity, adherence rates, concurrent nutritional interventions, baseline activity levels—may contribute to between-study variation.

#### 4.4.3 Interpretation

High heterogeneity combined with small sample sizes resulted in very low certainty evidence (GRADE) for physical function outcomes due to inconsistency and imprecision. True effects on muscle strength, sit-to-stand performance, and other functional measures remain uncertain. In contrast, relatively low heterogeneity for VO_2max_ (I^2^ = 31%) and consistency across subgroups support robust cardiorespiratory improvements.

Sensitivity analyses showed most pooled estimates remained stable after sequential study exclusion, except one influential study substantially affected walking ability results. This underscores the need for larger trials to confirm borderline functional mobility effects.

Future trials should prioritize standardized outcome assessment protocols, detailed HIIT prescription reporting (including time at target intensities), and comprehensive documentation of adherence and concurrent interventions.

The high heterogeneity observed for physical function outcomes (I^2^ = 75–94%) likely reflects differences in: (1) measurement methods (handgrip vs. isokinetic vs. 1-RM); (2) HIIT modalities (cycling vs. treadmill vs. Nordic walking); (3) test protocols (30-s chair stand vs. 5-repetition test); and (4) population characteristics (age 44–81 years; healthy vs. diabetic). These pooled estimates should be interpreted as average effects across heterogeneous conditions rather than precise estimates for specific settings.

### 4.5 Limitations

Beyond heterogeneity issues, several methodological considerations warrant attention. The limited number of studies per outcome (fewer than 10) precluded meta-regression analyses. Qualitative examination suggests the high heterogeneity in physical function outcomes (I^2^ = 75–89%) may reflect protocol differences, with HIIT combined with resistance training showing greater strength benefits than aerobic HIIT alone. First, most studies had short intervention durations (median 12 weeks), potentially insufficient for detecting changes in vascular function. While short-term HIIT (2 weeks) rapidly improves cardiopulmonary function, macrovascular and microvascular function remained unchanged, suggesting vascular adaptations may require longer periods (>12–24 weeks) (Klonizakis et al., 2014). Second, most studies used cycle ergometry, with limited data on other modalities. Third, we did not assess quality of life, psychological wellbeing, or long-term adherence patterns. Fourth, potential publication bias cannot be ruled out. The limited number of studies precluded meta-regression to formally explore sources of heterogeneity.

### 4.6 Future research directions

Future studies should prioritize adequately powered randomized controlled trials comparing different HIIT protocols with moderate-intensity training and combined training. Standardized reporting of exercise dose (intensity, duration, time near VO_2max_), adherence, and adverse events would enable precise dose-response modeling (Papadopoulou, 2020; MacInnis and Gibala, 2017). Longer follow-up is needed to determine whether VO_2max_ improvements translate into reduced cardiovascular events, disability, and mortality. Combining physiological and imaging measures with functional and patient-reported outcomes could clarify mechanisms linking HIIT to muscle, adipose tissue, and cardiometabolic health. Pragmatic studies are warranted to evaluate HIIT integration into community and clinical programs, including strategies to maximize adherence addressing established correlates of physical activity participation (Trost et al., 2002).

### 4.7 Conclusion

This systematic review provides high-quality evidence that HIIT elicits clinically meaningful improvements in cardiorespiratory fitness in middle-aged and older women with a time-efficient format. Current evidence does not support meaningful benefits of HIIT alone for muscle strength or physical function, outcomes central to sarcopenia prevention and disability reduction. HIIT should be incorporated as one element within comprehensive exercise programs including resistance and balance training to address the multidimensional needs of aging women.

## 5 Conclusion

This systematic review and meta-analysis provides high-certainty evidence that HIIT produces a significant improvement in maximal oxygen uptake in middle-aged and older women. In contrast, evidence for improvements in muscle strength and physical function is limited and of low to very low certainty. Current findings do not support the use of HIIT as a standalone intervention to address age-related declines in physical function. HIIT should be incorporated into multimodal exercise programs that include resistance and balance training to achieve broader functional benefits in this population.

## Data Availability

The original contributions presented in the study are included in the article/Supplementary Material, further inquiries can be directed to the corresponding author.
