# Supplementary material for: Effects of high intensity interval training (HIIT) on cardiopulmonary fitness and physical function in middle-aged and elderly women: a systematic review and meta-analysis
Source: Front Physiol. 2026 Mar 4;17:1778052. doi: 10.3389/fphys.2026.1778052 (PMC12995631; doi:10.3389/fphys.2026.1778052)
Supplement: Supplementary file 1 [file DataSheet1.pdf]

Supplementary Material

1 Supplementary Figures and Tables

Table S1

Data base	Term	Quantity
PubMed	("high-intensity interval training"[Title/Abstract]OR "high intensity interval training"[Title/Abstract]OR HIIT[Title/Abstract]OR "sprint interval training"[Title/Abstract])AND("middle aged"[Title/Abstract]OR "middle age "[Title/Abstract]OR "middle-aged"[Title/Abstract]OR "older adult*"[Title/Abstract]OR elderly[Title/Abstract]OR aged[Title/Abstract]OR postmenopausal[Title/Abstract])AND(female[Title]OR women[Title]OR woman[Title])	47
Web Of science	("high-intensity interval training" OR "high intensity interval training" OR HIIT OR "sprint interval training") (Abstract) and (middle-aged OR "middle age" OR "middle-aged" OR "older adult*" OR elderly OR aged OR "postmenopausal") (Abstract) and ("female" OR "women" OR "woman") (Title)	175
SCOPUS	(TITLE-ABS-KEY (high-intensity interval training) OR TITLE-ABS-KEY (high intensity interval training) OR TITLE-ABS-KEY (HIIT) OR TITLE-ABS-KEY (sprint interval training) AND TITLE-ABS-KEY (middle-aged) OR TITLE-ABS-KEY (middle age) OR TITLE-ABS-KEY (middle-aged) OR TITLE-ABS-KEY (female) OR TITLE-ABS-KEY (women) OR TITLE-ABS-KEY (woman)) AND (EXCLUDE (EXACTKEYWORD , "Human") OR EXCLUDE (EXACTKEYWORD , "Male") OR EXCLUDE (EXACTKEYWORD , "Humans") OR EXCLUDE (EXACTKEYWORD , "Adult") OR EXCLUDE (EXACTKEYWORD , "Female") OR EXCLUDE (EXACTKEYWORD , "Animal Experiment") OR EXCLUDE (EXACTKEYWORD , "Nonhuman") OR EXCLUDE (EXACTKEYWORD , "Animal") OR EXCLUDE (EXACTKEYWORD , "Animals") OR EXCLUDE (EXACTKEYWORD , "Animal Tissue") OR EXCLUDE (EXACTKEYWORD , "Rat") OR EXCLUDE (EXACTKEYWORD , "Rats") OR EXCLUDE (EXACTKEYWORD , "Animal Model") OR EXCLUDE (EXACTKEYWORD , "Mouse") OR EXCLUDE (EXACTKEYWORD , "Mice") OR EXCLUDE (EXACTKEYWORD , "Rats, Sprague-dawley") OR EXCLUDE (EXACTKEYWORD , "Animal Cell") OR EXCLUDE (EXACTKEYWORD , "Wistar Rat") OR EXCLUDE (EXACTKEYWORD , "Students") OR EXCLUDE (EXACTKEYWORD , "Mice, Inbred C57bl") OR EXCLUDE (EXACTKEYWORD , "C57bl Mouse") OR LIMIT-TO (EXACTKEYWORD , "High-intensity I	72

	nterval Training"))	

1.1 Supplementary Figures

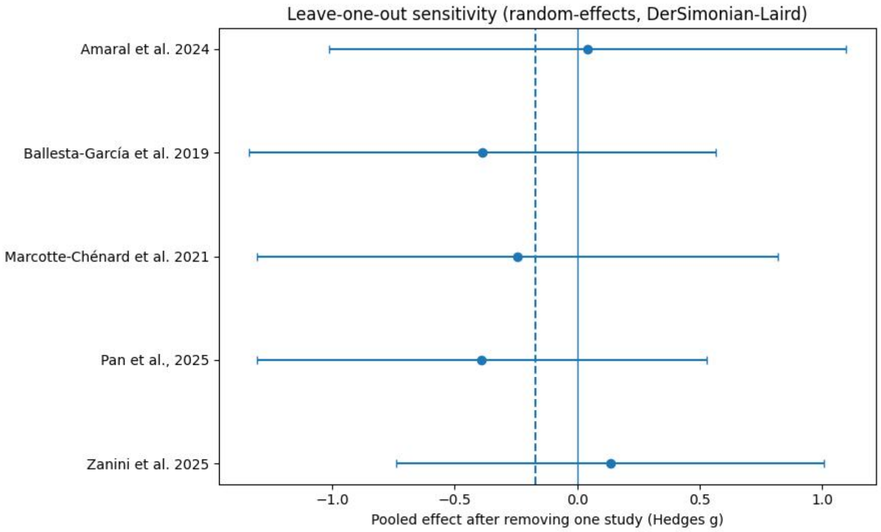

Supplementary Figure 1. Sensitivity analysis of skeletal muscle strength performance

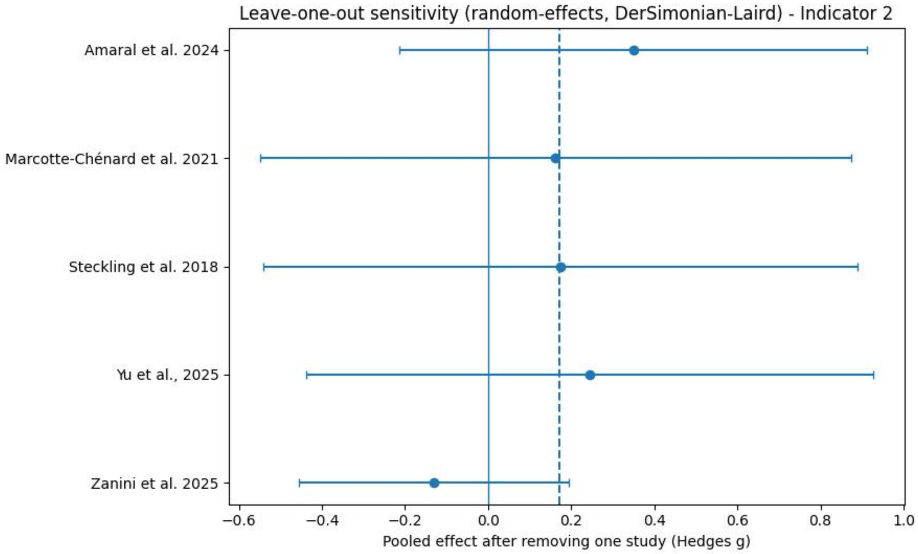

Supplementary Figure 2. Sensitivity analysis of flexibility performance

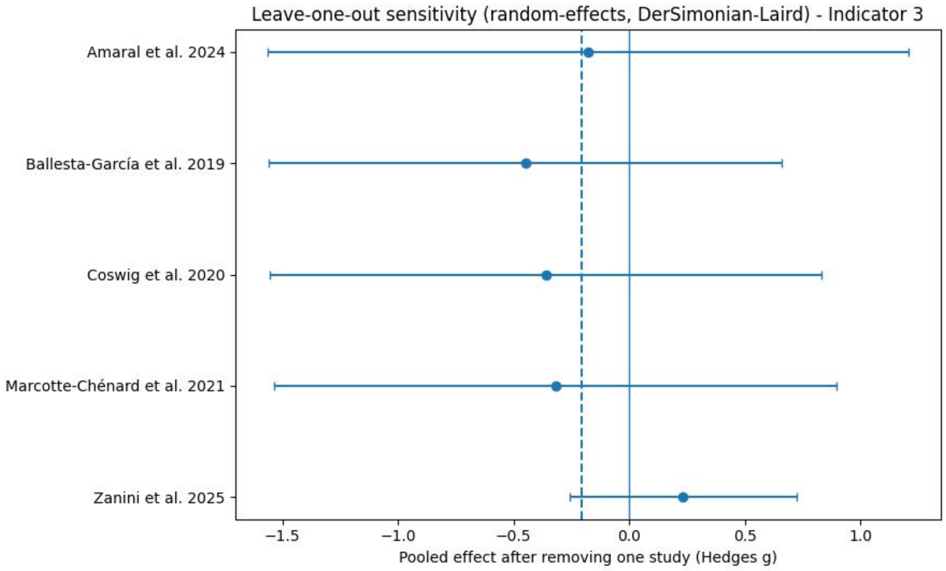

Supplementary Figure 3. Sensitivity analysis of standing ability

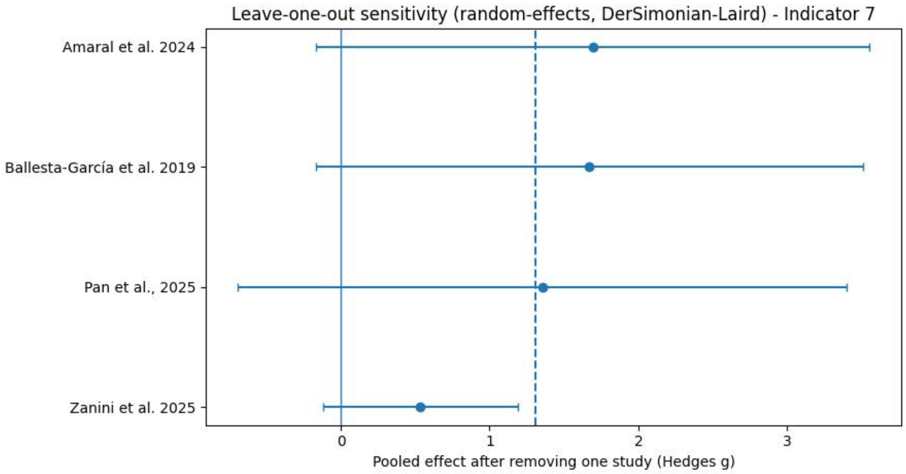

Supplementary Figure 4. Sensitivity analysis of Stand-up-walk test

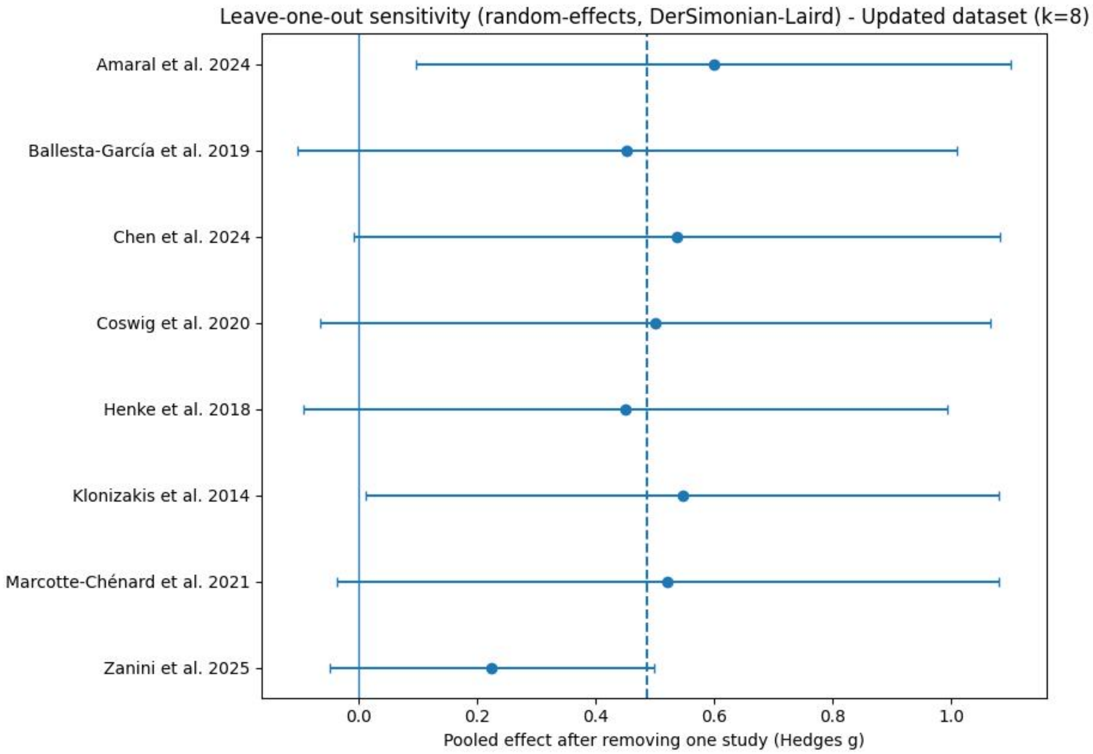

Supplementary Figure 5.. Sensitivity analysis of walking ability
